# Full vaccination coverage among children aged 12–23 months in Ethiopia: a systematic review and meta-analysis

**DOI:** 10.1186/s12889-020-08940-x

**Published:** 2020-05-24

**Authors:** Daniel Bekele Ketema, Moges Agazhe Assemie, Alehegn Aderaw Alamneh, Muluneh Alene, Kassahun Yawukal Chane, Yoseph Merkebe Alamneh, Molla Yigzaw Birhanu, Animut Alebel

**Affiliations:** 1 Department of Public Health, College of Health Sciences, Debre Markos University, P.O. Box 269, Debre Markos, Ethiopia; 2 Department of Human Nutrition and Food Science, College of Health Sciences, Debre Markos University, Debre Markos, Ethiopia; 3 School of Public Health, College of Medicine and Health Science, Hawassa University, Hawassa,, Ethiopia; 4 Deprtment of Biomedical Science, School of Medicine, Debre Markos University, Debre Markos, Ethiopia; 5 College of Health Sciences, Debre Markos University, Debre Markos, Ethiopia; 6 Department of Nursing, College of Health Sciences, Debre Markos University, Debre Markos, Ethiopia; 7 Faculty of Health, University of Technology Sydney, Sydney, Australia

**Keywords:** Vaccination coverage, Systematic review, Meta-analysis, Ethiopia

## Abstract

**Background:**

Vaccination is one of the most cost-effective means of public health interventions to prevent childhood deaths from infectious diseases. Although several fragmented studies have been conducted concerning full vaccination coverage among children aged 12–23 months in Ethiopia, the pooled estimate has not been determined so far. Therefore, this systematic review and meta-analysis aims to estimate the pooled prevalence of full vaccination coverage among children aged 12–23 months in Ethiopian.

**Methods:**

To find potentially relevant studies, we systematically searched five major databases (i.e., PubMed/MEDLINE, CINAHL, EMBASE, Google Scholar, and Science Direct). This review included community based cross-sectional studies reported in English language; had good quality, and published from the 1st of January 2000 to the 20th of November 2019. Data were analyzed using Stata™ Version 14.1 software. The pooled estimates with 95% confidence intervals (CIs) were presented using forest plots. Higgins and Egger’s tests were used to assess heterogeneity and publication bias, respectively. Primary estimates were pooled using a random effects meta-analysis model.

**Results:**

Of the total of 851 identified articles 21 studies involving 12,094 children met the inclusion criteria and were included in this meta-analysis. The included studies sample size ranged from 173 to 923. The lowest proportion of full vaccination coverage was reported from Afar Region [21% (95% CI: 18, 24%)], whereas the highest proportion of full vaccination coverage was reported from Amhara Region [73% (95% CI: 67, 79%)]. The overall prevalence of full vaccination coverage among children in Ethiopia was 60% (95% CI: 51, 69%).

**Conclusions:**

Our finding suggested that six in every 10 children in Ethiopia were fully vaccinated. However, this finding is much lower than the World Health Organization recommended rate. Moreover, high regional variations in terms of full vaccination coverage across the country was observed. Therefore, a special attention should be given to improve the overall childhood vaccination coverage.

## Background

Although the world made remarkable progress in reducing under-five mortality from 12.6 million deaths in 1990 to 5.4 million in 2017, it remains a serious public health problem [1]. In 2017, an estimated 5.4 million children under the age of five died worldwide. This translates into 15,000 deaths per day. Sub-Saharan Africa (SSA) continues to be the region with the highest under-five mortality rate (76 deaths per 1000 live births in 2017) in the world [1, 2]. According to the 2019 Ethiopian Mini Demographic and Health Survey (EMDHS) report, under-five mortality in Ethiopia was 55 deaths per 1000 live births [3]. More than half of early childhood deaths are due to diseases that could be easily prevented or treated with simple and affordable interventions, such as administering vaccines [2, 4].

Vaccination is one of the most cost-effective means of public health interventions to prevent deaths from childhood infectious diseases. Currently, vaccination prevents 2–3 million deaths annually. An additional 1.5 million deaths could be totally avoided through vaccination [5]. In developing countries, about 16% of under-five deaths are attributed to vaccine-preventable diseases [6]. In Ethiopia, vaccine-preventable diseases such as pneumonia and diarrheal disease are the leading causes of under-five mortality [7]. Despite the benefits above-mentioned, approximately 19.4 million infants worldwide were not reached by immunization services in 2018. The total number of unvaccinated children, 60% lived in 10 countries: Angola, Brazil, the Democratic Republic of the Congo, Ethiopia, India, Indonesia, Nigeria, Pakistan, the Philippines, and Vietnam [8]. According to the 2019 EMDHS report, only 43% of Ethiopian children aged 12–23 months were fully vaccinated [3].

The World Health Organization (WHO) launched the Expanded Programme on Immunization (EPI) in 1974, intending to provide universal access to all relevant vaccines for all at risk [9]. EPI in Ethiopia was started in 1980, with a plan to reach 100% coverage in 1990 [10]. The Ethiopian government mobilized the volunteer Women’s Development Army or volunteers, health extension workers (HEWs), and health facilities to achieve universal immunization coverage [7, 11]. likewise, the immunization coverage in Ethiopia increased from 14.3% in 2000 to 43% in 2019 [3, 12]. To improve vaccination coverage by implementing different effective interventions, comprehensive nationwide evidence is vital. In Ethiopia, despite many fragmented studies that have been reported so far, a study representing the national and regional immunization coverage is lacking. The reasons mentioned above triggered us to conduct this comprehensive review to summarize the available evidence on routine immunization in Ethiopia. Thus, this review is intended to estimate the national coverage of childhood immunization in Ethiopia. Results obtained from this review will help health policymakers to design evidence-based public health responses.

## Methods

### Data source and searches

This review was reported according to the Preferred Reporting Items for Systematic Review and Meta-Analysis (PRISMA) guideline [13] (Supplementary 1). To find potentially relevant studies, we systematically searched five major databases (i.e., PubMed/MEDLINE, CINAHL, EMBASE, Google Scholar, and Science Direct). Additionally, the reference lists of eligible studies were checked for additional articles. The search was conducted by two authors (DBK and AA) independently. Studies identified through systematic search were retrieved and managed using Endnote X7. The search from the above mentioned databases was conducted using the following terms: “immunization” OR “vaccination” AND “children” OR “childhood” AND “Ethiopia”. The search was started in September 2019.

### Study selection criteria

#### Inclusion Criteria

**Design:** Community based cross-sectional studies

**Study setting:** Ethiopia

**Population:** Children aged 12–23 months

**Publication status:** All published and unpublished articles

**Language:** English language

**Publication date:** Published from the 1st of January 2000 to the 20th of November, 2019

#### Exclusion criteria

Articles that were not fully accessed after at least two email contacts of the principal investigator were excluded.

### Screening process

We included all community based cross-sectional studies. All titles/abstracts identified in the electronic databases were screened by 2 authors (DBK, AA) independently of one another. Discrepancies were resolved by discussion. All potentially relevant full texts were screened by 2 authors (DBK, AA) independently of one another. Discrepancies were resolved by discussion. In the case of discrepant judgements, a third author (AAA) was involved.

### Data extraction process and quality assessment

Six authors (AAA, MAA, KYC, MA, MYB, and YMA) independently performed data extraction using a pre-defined eligibility criterion to ensure consistency. The data extraction form was prepared using a Microsoft™ Excel spreadsheet. Disparities between authors were resolved through discussion once the source of disagreements were identified. The following information were extracted from each primary article: number of children with full vaccination, proportion of full vaccination, study location, region, publication year, study design, sample size, and first author (Supplementary file 2).

The quality of included studies was appraised using the Newcastle-Ottawa Quality assessment scale (NOQAS) [14]. The quality of each study was assessed using the following criteria: representativeness of the study, adequate sample size, acceptable non-response rate, used validated measurement tool, comparability of the study, description of outcome assessment, and used appropriate statistical tests. Articles with a global rating score ≥ seven out of 10 were considered to be high quality [15, 16] (Supplementary file 3).

### Outcome variable

Full vaccination coverage (%) was the primary outcome measure of this study. According to WHO, a child is considered fully vaccinated after receiving (i) one dose of Bacille Calmette-Guerin (BCG); (ii) three doses of oral polio vaccine (OPV); [diphtheria, pertussis (whooping cough), and tetanus (DPT)], hepatitis B vaccines (HBV), and (iii) one dose of measles vaccine; all before attaining 1 year [17, 18]. We included studies that fulfilled the above definition. However, we found studies included other vaccines such as rota virus, pneumococcal conjugate, and Hib in their immunization schedules. In such case, we carefully checked the compliance with the above mentioned case definition before considering for our analysis.

### Heterogeneity and publication bias

The presence of statistical heterogeneity within the included studies was checked using I-square statistics and Cochran’s-Q test. Accordingly, heterogeneity was classified as low, moderate, or high when the values of I-square were 25, 50, and 75%, respectively [19]. Additionally, the dispersion of individual results in the forest plot was also used to evaluate the presence of heterogeneity visually. Egger’s weighted regression test at a *p*-value < 0.05 was used to assess the presence of publication bias [20].

### Data synthesis

Relevant data from each primary study were extracted using a Microsoft™ Excel form. Then, the data were exported to Stata™ Version 14.1 software for further analysis. The overall pooled estimate was computed using *metaprop* stata command. The standard errors were calculated from the reported estimates and population denominators using a binominal distribution assumption. A random effects meta-analysis model was computed using the DerSimonian and Laird Method [21]. Further statistical analyses such as subgroup analyses, meta-regression were performed to identify the possible sources of heterogeneity. We performed a subgroup analysis based on geographical regions of the country. Furthermore, sensitivity analysis using a random effects model was performed to assess the influence of a single study on the overall pooled estimate. At last, results were presented in tables and forest plots.

## Results

### Search results and study selection

**Fig. 1:**
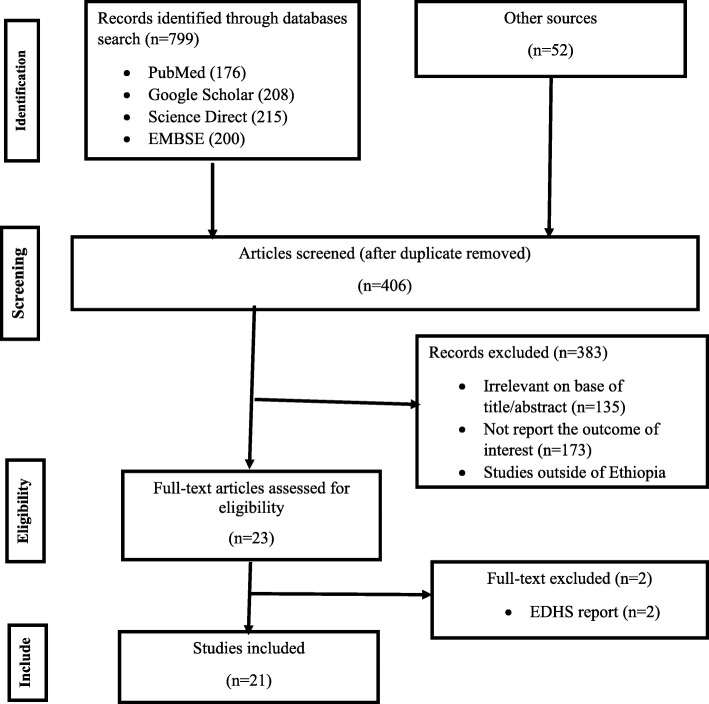
PRISMA flow diagram which shows the selection of included studies for full vaccination coverage among children aged 12–23 months in Ethiopia, from 2000 to 2019

The online search yielded 851 results. Then, after removal of duplicates using EndNote × 7, studies were screened for title and abstract. a. Finally, 23 full texts were retrieved and downloaded to be assessed using our inclusion criteria. Reasons for exclusion of articles were outlined in Fig. 1. From these 23 full-texts, two EDHS based articles were excluded [22, 23]. Because EDHS reports were crude and governmental surveys and due to political instability the report may produce poor quality evidence. In addition, owing to instability in different regions of the country, particular regions were excluded from the survey by the government. As a result, EDHS reports lacks representativeness.

### Characteristics of eligible studies

As presented in Table 1, 21 studies met the inclusion criteria and were included. All the included articles were community based cross-sectional studies. All included studies were published between January 2000 and November 2019. The median year of publication was 2015. A total of 12,094 (mean = 576 children) children were included in this review. Of these 21 studies included in the meta-analysis, nine reported the mean age of the study children [24, 25, 28, 30, 31, 33, 34, 39, 42]. Based on the nine studies, the mean age ranged from 16.39 [31, 42] to 18 [34] months.

**Table 1:** Descriptive summary of 21 included studies in the systematic review and meta-analysis of full immunization coverage among children aged 12–23 months in Ethiopia, from 2000 to 2019

First Author	Year	Location (Region)	Study area	Mean age (Months)	Sample size	Coverage of fully Vaccination (%)
Debie A [24]	2014	Amhara	Mecha District	17.5	497	49.3
Girmay A [25]	2019	Amhara	Sekota Zuria	16.7	620	77.4
Mohamud AN [26]	2014	Somali Region	Jigjiga	–	582	36.6
Tenaw G [27]	2017	Amhara	Debre Markos	–	288	91.7
Hailu S [28]	2019	SNNPR	Wonago	–	923	63.4
Tolera D [29]	2014	Addis Ababa	Addis Ketema sub-city	–	585	72.4
Legesse E [30]	2015	Oromia	Sinana District	17.9	591	76.8
Tesfaye TD [31]	2018	Amhara	East Gojjam	16.39	830	58.4
Okwaraji YB [32]	2012	Amhara	Dabat District	–	775	81.7
Etana B [33]	2012	Oromia	Ambo Woreda	16.8	536	36.0
Meleko A [34]	2017	SNNPR	Bench Maji Zone	18	322	42.2
Ayano B [35]	2015	SNNPR	Hosanna Town	–	508	30.5
Fite RO [36]	2019	SNNPR	Areka Town	–	173	75.1
Kassahun MB [37]	2015	Amhara	Lay Armachiho District	–	751	76.0
Mekonnen AG [38]	2019	Amhara	Minjar-Shenkora District	–	566	75.6
Animaw W [39]	2014	SNNPR	Arba Minch Town	17.4	630	73.2
Ebrahim T [40]	2015	Amhara	Tehuledere District	–	539	83.1
Lake MW [41]	2015	Amhara	Dessie Town	–	724	65.2
Mohammed H [42]	2013	Oromia	East Hararghe Zone	16.39	694	22.9
Kidane T [43]	2000	Tigray	Mekele	–	220	51.0
Beyene E [44]	2013	Afar	Afar,Zone 3	–	740	20.6

*SNNPR* South Nations and Nationalities People of the Region

The smallest (*n* = 173) and largest (*n* = 923) sample sizes were reported from studies done in the Southern Nation’s, Nationalities’, and People’s Region (SNNPR) [28, 36]. Likewise, the lowest (20.6%) vaccination coverage was reported from Afar Region [44] whereas the highest (83.1%) prevalence of vaccination coverage was reported from Amhara Region [40]. Geographically, nine studies were undertaken in Amhara Region [24, 25, 27, 31, 32, 37, 38, 40, 41], five in the SNNPR [28, 34–36, 39], three in Oromia Region [30, 33, 42],and one in each in Tigray Region [43], Addis Ababa [29], Somali Region [26], and Afar Region [44]. However, we did get studies from Benishangul Gumuz Region, Dire-Dawa City Administration, Harari Region, and Gambella Region. This showed that majority of the researches were undertaken in Amhara Region. The quality score of included studies ranged from eight to 10, with a mean quality score of 9.71 (SD ± 0.421) (Supplementary file 3). Overall, studies with a quality score of ≥ seven were considered as high-quality. Lastly, all the 21 included articles were categorized as high-quality studies [15, 16].

### Pooled full vaccination coverage in Ethiopia

**Fig. 2:**
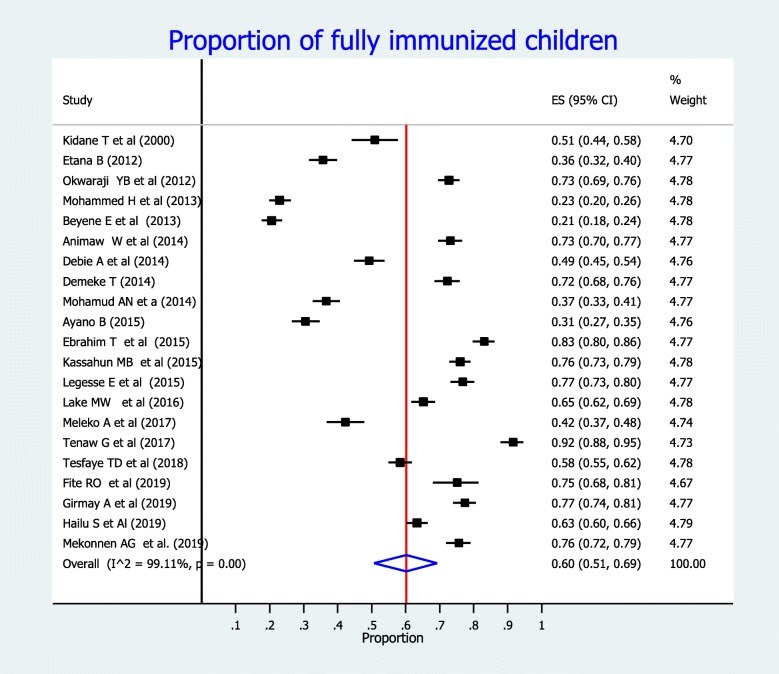
Forest plot showing the proportion of full vaccination coverage among children aged 12–23 months in Ethiopia, from 2000 to 2019

**Fig. 3:**
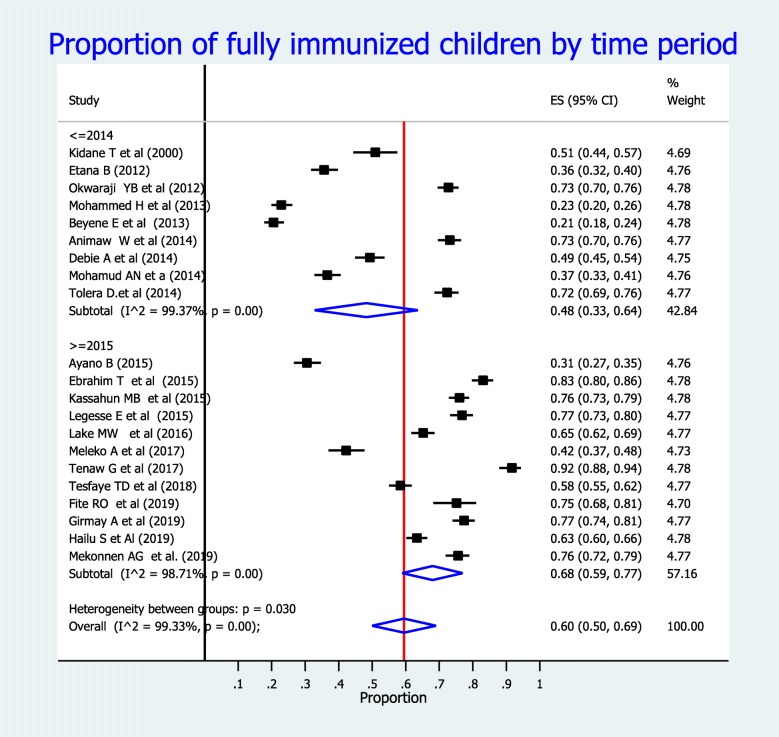
Forest plot showing the proportion of full vaccination coverage among children aged 12–23 months in Ethiopia, from 2000 to 2019 by time period

The overall pooled estimate of full vaccination coverage among children aged 12–23 months in Ethiopia was found to be 60% (95% CI: 51, 69%) (Fig. 2). Regional subgroup analyses revealed that the highest (73, 95% CI: 67, 79%) proportion of full vaccination coverage was found in Amhara Region; followed by Addis Ababa (72, 95% CI: 69, 76%), and SNNPR (53, 95% CI: 33, 72%). Conversely the lowest (21, 95% CI: 18, 24%) vaccination coverage was observed in Afar Region (Table 2). As shown in Fig. 3, the proportion of full vaccination coverage among children before 2015 was 48% (95% CI: 33, 64%), whereas it was 68% (95% CI: 59, 77%) after 2015.

**Table 2:** Subgroup analysis of full vaccination coverage among children aged 12–23 months by region in Ethiopia, from 2000 to 2019

S. No	Region	Estimate of full vaccination coverage [% (95% CI)]
1	Amhara	73 (67, 79),
2	Oromia	28 (25, 30)
3	SNNPR	53 (33, 72)
4	Somali regional state	37 (33, 41)
5	Addis Ababa	72 (69,76)
6	Tigray	51 (44, 57)
7	Afar	21 (18, 24)
8	Overall pooled estimate	60 (51, 69)

*SNNPR* South Nations and Nationalities People of the Region

### Meta regression and publication bias

**Fig. 4:**
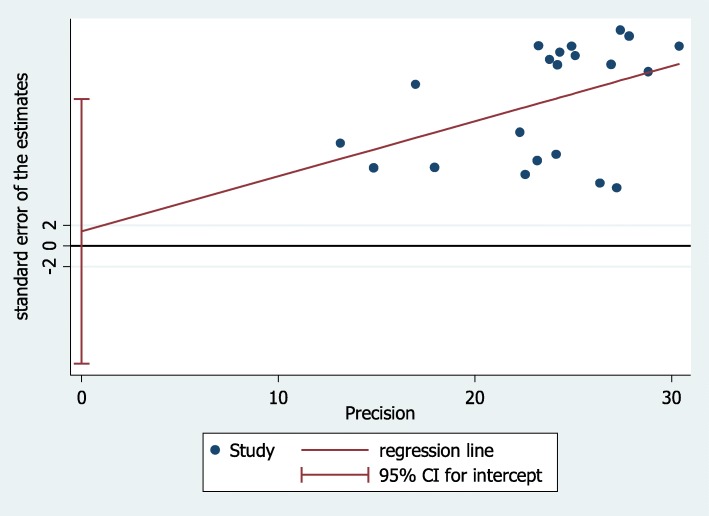
Funnel plot to check publication bias of the full vaccination coverage among children aged 12–23 months in Ethiopia by sample size, and publication year of included studies

Random-effects meta-regression was conducted by considering year of publication and sample size as covariates. The analysis indicated that heterogeneity was not explained by sample size (*p* = 0.443) and publication year (*p* = 0.117) (Table 3). Funnel plot asymmetry was assessed using Egger’s weighted regression test to examine the presence of publication bias. However, no statistically significant of publication bias was detected (*p* = 0.822) (Fig. 4).

**Table 3:** Meta-regressions of the full vaccination coverage among children aged 12–23 months in Ethiopia by sample size, and publication year of included studies

Covariate	β (95% CI)	*P*-Value
Publication year	0.019 (− 0.005, 0.043)	0.117
Sample size	−0.00019 (− 0.00069, .000319)	0.443

## Discussion

To the best of our knowledge, this systematic review and meta-analysis is the first of its kind to estimate the full vaccination coverage among children aged 12–23 months in Ethiopia. The overall pooled proportion of full vaccination coverage among children in Ethiopia was found to be 60% (95% CI: 50, 69%). This finding is in agreement with the findings of studies conducted in Kenya (57.7%) [45], Malawi (51%) [46], and Uganda (68%) [47]. However, our finding is higher than the vaccine coverage proportions presented in the 2011 EDHS (24%), the 2016 EDHS (39%), and the 2019 EMDHS (43%) reports [3, 23, 48]. The above differences could be elucidated by the fact that the demographic and health surveys were conducted in different segments of the country; which contained data from children live in rural and urban areas. However, in our meta-analysis only community based cross-sectional studies were included. Similarly, our estimate is also higher compared to reports from Nigeria (34.4%) [49], India (39%) [50], and Brazil (47%) [51]. These discrepancies might be due to differences in data generating methods, and the level of government interventions and commitments.

On the other hand, our finding is much more lower than the WHO recommended level (≥ 90%) [52]. This shortfall in reaching WHO’s 90% target could be due to common challenges facing the immunization program in Ethiopia, such as immunization service interruption due to supply shortages, limited outreach services in hard-to-reach communities, and EPI staff turnover [53].

The subgroup analyses also showed that vaccination coverage across regions of Ethiopia was highly dispersed. The lowest coverage was observed in Afar Region (21%), while the highest coverage was observed in Amhara Region (73%). This regional variation is in line with the 2019 EMDHS report [3]. This discrepancy could be explained by differences in the caregiver’s educational level as well as differences in socio-cultural and religious backgrounds.

Additionally, the pooled estimate of full vaccination coverage before 2015 was 48%, whereas the pooled estimate of full vaccination coverage after 2015 was 68%. From this finding, we can understand that the proportion of vaccine coverage among children has increased slightly in every consecutive year. This finding implies that the country has been implementing different strategies to improve childhood vaccination coverage. This finding is in parallel with the EDHS surveys conducted over time indicated that the vaccination coverage has increased from 14.4% in 2000 to 43% in 2019 [3, 12]. This promising increase in the proportion of full vaccination coverage might be due to improvements in accessibility and provision of immunization services to the wider population. However, since the inclusion period is almost 20 years, children born in 2000 are now adults; therefore, their vaccination coverage may not represent the current childhood vaccination coverage.

Since this included community based cross-sectional studies, the findings can be generalized to the entire population of children aged 12–23 months in Ethiopia. However, our review has some limitations. Firstly, we were unable to find studies conducted in some regions of the country. Therefore, further community-based studies shall be done in regions such as Benishangul Gumuz, Dire-Dawa City Administration, Harari, and Gambella. Such that not having estimates for them might bias pooled coverage estimates. Since these regions were found far away from the central location of the country and presence of some insurrection, may contribute for lack of evidences for our estimate. Secondly, the current review considered only papers published in English language. At last, some biases might be introduced sine we used the NOS for quality assessment tool. In this tool, some domains were not univocal; and lacked comprehensive definition for each domain.

## Conclusions

Our review suggested that six in every 10 children in Ethiopia were fully vaccinated. However, this finding is much lower than the WHO-recommended level (≥ 90%). In addition, vaccination coverage among children was highly varied across the regions of the country. Furthermore, in Ethiopia, the vaccine coverage among children has increased slightly in each consecutive year.

Therefore, a special attention should be given to improve the overall childhood vaccination coverage across the country.

## Supplementary information

**Additional file 1.** PRISMA 2009 checklist.

**Additional file 2.** Data extraction sheet.

**Additional file 3.** Newcastle-Ottawa Quality assessment scale (NOQAS).

## Abbreviations

CI: Confidence Interval
WHO: World Health Organization
MMR: Maternal Mortality Ratio
ANC: Antenatal Care
EPI: Expanded Programme on Immunization
EDHS: Ethiopia Demographic and Health Survey
EMDHS: Ethiopia Mini Demographic and Health Survey
UNICEF: United Nations Children’s Fund
SSA: Sub-Saharan Africa
